# Functional sympatholysis in mouse skeletal muscle involves sarcoplasmic reticulum swelling in arterial smooth muscle cells

**DOI:** 10.14814/phy2.15133

**Published:** 2021-12-01

**Authors:** Jennifer van der Horst, Sophie Møller, Sasha A. S. Kjeldsen, Jørgen F. P. Wojtaszewski, Ylva Hellsten, Thomas A. Jepps

**Affiliations:** ^1^ Department of Biomedical Sciences University of Copenhagen Copenhagen Denmark; ^2^ Department of Nutrition, Exercise and Sports The August Krogh Section for Human Physiology University of Copenhagen Copenhagen Denmark; ^3^ Department of Nutrition, Exercise and Sports The August Krogh Section for Molecular Physiology University of Copenhagen Copenhagen Denmark

**Keywords:** exercise, sarcoplasmic reticulum, sympathetic vasoconstriction, Sympatholysis, α‐adrenergic receptors

## Abstract

The vasoconstrictive effect of sympathetic activity is attenuated in contracting skeletal muscle (functional sympatholysis), allowing increased blood supply to the working muscle but the underlying mechanisms are incompletely understood. The purpose of this study was to examine α‐adrenergic receptor responsiveness in *isolated* artery segments from non‐exercised and exercised mice, using wire myography. Isometric tension recordings performed on femoral artery segments from exercised mice showed decreased α‐adrenergic receptor responsiveness compared to non‐exercised mice (logEC_50_ −5.2 ± 0.04 M vs. −5.7 ± 0.08 M, respectively). In contrast, mesenteric artery segments from exercised mice displayed similar α‐adrenergic receptor responses compared to non‐exercised mice. Responses to the vasoconstrictor serotonin (5‐HT) and vasodilator isoprenaline, were similar in femoral artery segments from non‐exercised and exercised mice. To study sarcoplasmic reticulum (SR) function, we examined arterial contractions induced by caffeine, which depletes SR Ca^2+^ and thapsigargin, which inhibits SR Ca^2+^‐ATPase (SERCA) and SR Ca^2+^ uptake. Arterial contractions to both caffeine and thapsigargin were increased in femoral artery segment from exercised compared to non‐exercised mice. Furthermore, 3D electron microscopy imaging of the arterial wall showed SR volume/length ratio increased 157% in smooth muscle cells of the femoral artery from the exercised mice, whereas there was no difference in SR volume/length ratio in mesenteric artery segments. These results show that in arteries surrounding exercising muscle, the α‐adrenergic receptor constrictions are blunted, which can be attributed to swollen smooth muscle cell SR’s, likely due to increased Ca^2+^ content that is possibly reducing free intracellular Ca^2+^ available for contraction. Overall, this study uncovers a previously unknown mechanism underlying functional sympatholysis.

## Graphical abstract

The mechanisms blunting sympathetic vasoconstriction in exercising muscle—termed functional sympatholysis—remain incompletely understood. We have taken a completely novel approach and studied femoral artery segments isolated from the skeletal muscle in mice immediately after exercise. This study reveals a previously unknown mechanism contributing to functional sympatholysis over 90 years after the phenomenon was first described.

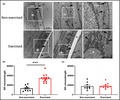

## 1 INTRODUCTION

During exercise, blood flow to the active muscle is enhanced to meet the increased metabolic demand. The increase in blood flow occurs despite an enhanced sympathetic nerve activity (SNA) and is achieved through a combination of vasodilators produced locally and a phenomenon termed “functional sympatholysis” (Rein, 1930; Remensnyder et al., 1962; Thomas & Segal, 2004). The mechanisms responsible for functional sympatholysis remain incompletely understood.

Increased intracellular calcium concentration ([Ca^2+^]_i_) is the principal trigger for smooth muscle contraction. SNA produces the release of catecholamines, including norepinephrine (NE). Following increased SNA, NE binds α_1_‐adrenergic receptors predominantly on vascular smooth muscle, which would normally lead to vasoconstriction elicited by increased [Ca^2+^]_i_, as well as activation of the Rho‐associated kinase calcium sensitization pathway (Aburto et al., 1993; Somlyo & Somlyo, 2003; Stull et al., 1991). However, in functional sympatholysis, arteries supplying exercising muscle are impervious to the increased SNA (and NE) and will dilate to ensure adequate blood flow to the working, metabolically active muscle.

Sympathetic vasoconstriction is blunted in the arteries and arterioles supplying muscles during exercise, which facilitates blood flow delivery to the exercising muscles. Several mechanisms are proposed to attenuate the sympathetic vasoconstriction, including increased wall shear stress leading to increased nitric oxide release from the endothelium, release of vasodilators from blood constituents, and release of vasodilative metabolites from contracting skeletal muscles (Clifford & Hellsten, 2004; Saltin & Mortensen, 2012; Thomas & Segal, 2004). As such, several mediators of functional sympatholysis have been shown experimentally, including ATP and nitric oxide, although the relative contribution of each these mediators remains unclear (Chavoshan et al., 2002; Clifford & Hellsten, 2004; Hearon et al., 2017; Saltin & Mortensen, 2012; Thomas & Segal, 2004). Upstream conduction of vasodilator responses from distal arterioles to feed arteries has also been shown to attenuate sympathetic vasoconstriction, thereby increasing muscle blood flow to meet the metabolic demands of the working muscle (Segal, 2005). These mechanisms are crucial for functional sympatholysis; however the observed sympatholytic responses cannot be attributed solely to the vasodilating actions of these mechanisms. Although these factors are capable of inducing vasodilatation, it is not completely understood whether they are also responsible for vascular adaptations during exercise to further attenuate sympathetic vasoconstriction in a single bout of exercise.

The present study aimed to assess α‐adrenergic receptor responsiveness in ex vivo femoral artery segments of non‐exercised and exercised mice. This approach allows examination of vascular reactivity without confounding changes in systemic hemodynamics and avoids continuous complex interplay between sympathetic vasoconstrictor outflow and vasoactive signals released locally from the muscle, thus providing the direct assessment of α‐adrenergic receptor responsiveness in isolated blood vessels. Furthermore, we utilized 3D electron microscopy of the vascular wall to visualize phenotypic histological adaptations of arteries supplying working muscle.

## 2 METHODS

### 2.1 Animals

All animal experiments were performed in accordance with Directive 2010/63EU on the protection of animals used for scientific purposes and approved by the national ethics committee, Denmark (approval #2019‐15–0201–01659) and conformed to the ARRIVE guidelines (Percie du Sert et al., 2020). Female C57Bl/6J mice, 10 weeks old, were purchased from Taconic Biosciences (Ejby, Denmark), group‐housed in clear plastic containers, maintained at 22–24°C on a 12‐h light/dark cycle with ad libitum access to water and rodent chow diet. The mice underwent at least 1 week of habituation and 1 week of treadmill acclimatization. Since female mice do not display the same variability in their daily activity pattern when housed together (Robbers et al., 2021), this study focused on female mice to avoid potential awake–sleep cycle variability on exercise performance.

### 2.2 Treadmill acclimatization and experimental treadmill running protocol

Animals were randomly subdivided into two groups (exercised and non‐exercised), with a sample size of 10 mice in each group. The exercised mice were acclimatized to treadmill running (TSE Systems GmbH; Homburg, Germany) prior to the experimental day. Acclimatization of the mice was performed on days 6, 5, 4, 3, and 2, with a rest on day 1. The acclimatization protocol consisted of a 10‐minute rest on the treadmill on day 6; while on days 5–3, acclimatization consisted of a 5‐minute rest on the treadmill followed by 5 min at a tempo of 0.084, 0.25, and 0.34 ms^−1^, respectively. On day 2, mice were subjected to a graded running protocol consisting of a 5‐minute rest followed by a 5‐minute run at 0.1 ms^−1^ and a gradual increase in speed by 0.01 ms^−1^ every 60 s. The runs were stopped when mice touched the electrical shock grid at the back of the treadmill three consecutive times or were unresponsive to puffs of air in a 60‐second period. All runs were performed on a 10‐degree incline (Jørgensen et al., 2005).

On the experimental day (day 0), the following protocol was performed on the exercising mice based on the average 60% maximal speed observed on day 2: first, a 5‐minute rest on the treadmill conveyor belt followed by 5 min of gradually increased speed to a final velocity of 0.15 ms^−1^ (60% maximal speed). The mice ran for 10 min at this final velocity. Non‐exercised control mice were in their cages in the same room as the treadmills. After the exercise was completed, the exercised and control mice, were sacrificed by cervical dislocation, and mesenteric and femoral arteries were removed for myography experiments and imaging.

### 2.3 Myography

After euthanasia, third‐order mesenteric arteries and the conduit femoral arteries from both hind limbs were dissected immediately and placed in ice‐cold physiological salt solution (PSS) containing (in mM): 121 NaCl, 2.8 KCl, 1.6 CaCl_2_, 25 NaHCO_3_, 1.2 KH_2_HPO_4_, 1.2 MgSO_4_, 0.03 EDTA, and 5.5 glucose. Following dissection, the arteries were cleaned of adherent tissue and perivascular adipose tissue. Arteries were cut into 2 mm segments, and mounted on 40 µm stainless steel wires in a wire myograph (Danish Myo Technology, Aarhus Denmark) for isometric tension recordings. Each artery segment was used for one functional experiment. The chambers of the myograph contained PSS maintained at 37°C and aerated with 95% O_2_/5% CO_2_. The changes in tension were recorded continuously by PowerLab and Chart software (ADInstruments, Oxford, United Kingdom). Arterial segments were equilibrated for 30 min and normalized to passive force (Mulvany & Halpern, 1977). Subsequently, the PSS was replaced with a high potassium solution (KPSS) containing (in mM): 123.7 KCl, 1.6 CaCl_2_, 25 NaHCO_3_, 1.2 KH_2_HPO_4_, 1.2 MgSO_4_, 0.03 EDTA, and 5.5 glucose to assess viability. The chambers were washed with PSS before artery segments were constricted with sequentially increasing concentrations of the α1‐adrenoceptor agonist, methoxamine (0.1–30 µM) or serotonin (5‐HT, 0.01–3 µM). Furthermore, as NE may also bind to β‐adrenergic receptors and cause vasodilation that could possibly oppose the SNA‐mediated vasoconstriction (Kneale et al., 2000), we investigated whether exercise increased β‐adrenoceptor sensitivity by applying a β‐adrenergic receptor agonist, isoprenaline (0.001–3 µM), cumulatively to pre‐contracted (10 μM methoxamine) arteries. To assess sarcoplasmic reticulum function, vessels were treated with 10 mM caffeine or 1 μM thapsigargin, and tension was expressed as a percentage of the steady‐state tension (100%) obtained with KPSS.

### 2.4 Electron microscopy

Small sections (<1 mm) of conduit femoral and third‐order mesenteric arteries were fixed by immersion in 2% v/v glutaraldehyde in 0.05 M sodium phosphate buffer (pH 7.4), rinsed three times in 0.15 M sodium cacodylate buffer (pH 7.4), and subsequently stained according to Ellisman (https://ncmir.ucsd.edu/sbem‐protocol). Briefly, the samples were post‐fixed in 1% w/v osmium tetroxide (OsO_4_) and 0.05 M potassium ferricyanide in 0.12 M sodium cacodylate buffer (pH 7.4) for 2 h. After washing with H_2_O, the samples were incubated in freshly prepared 1% thiocarbohydrazide solution in H_2_O for 20 min at RT. The samples were then rinsed and further stained with 2% OsO_4_ in H_2_O for 30 min at RT. Subsequently, the samples were rinsed and *en* *bloc* stained with 1% uranyl acetate in H_2_O overnight at RT. In the final step of staining, the specimens were rinsed and treated *en* *bloc* with Walton's lead aspartate for 30 min at 60°C. Following several washes, the specimens were dehydrated in graded series of ethanol, transferred to propylene oxide, and embedded in Epon according to the standard procedures. After resin polymerization, a 300 × 300 μm mesa including the region of interest was trimmed with an ultramicrotome (Leica EM UC7, Leica Microsystems). The resin block was mounted on a stub and immobilized using Epo‐Tek EE129‐4 Adhesive (EMS #12670‐EE) and subsequently sputter coated with 20 nm of gold (ACE200, Leica Microsystems). The 40 nm serial images (2 × 2 k, 10 nm pixel resolution) were collected by a serial block face‐scanning electron microscope (SEM), which was equipped with an in‐chamber diamond knife, (Teneo VolumeScope II, FEI Company) using back scattered electron signals at an accelerating tension of 1.78 kV under high vacuum conditions. The SEM images were imported into the Amira software (Thermo Fisher Scientific) for 3D visualization and data analysis. A segmentation tool was used to determine the 3D structure of the SR, after which volumetric analysis was applied to determine volume/length ratio for individual SRs. SR volume/length was analyzed in at least two different vascular smooth muscle cells per vessel segment. The analysis was carried out unblinded.

### 2.5 Statistical analysis

For myography experiments, the n values represent data from a single vessel segment taken from separate mice. For EM, three control mice and three exercised mice were investigated for changes in SR volume. The n values represent single SR volume/length ratios measured in different regions of the arterial walls. GraphPad Prism 8 was used for statistical analysis. LogEC_50_ values for concentration responses were determined from individual experiments by fitting data to a four‐parametric nonlinear regression analysis (bottom/hillslope/top/EC_50_). Mean logEC_50_ and maximum relaxation (Rmax) values from individual experiments were compared by an unpaired *t* test. Mean SR volume/length ratios were compared with an unpaired *t* test. All data are presented as mean ± standard error of the mean (SEM). Significance at the *p* ≤ *0*.*05* level is denoted in all figures with *.

## 3 RESULTS

**FIGURE 1:**
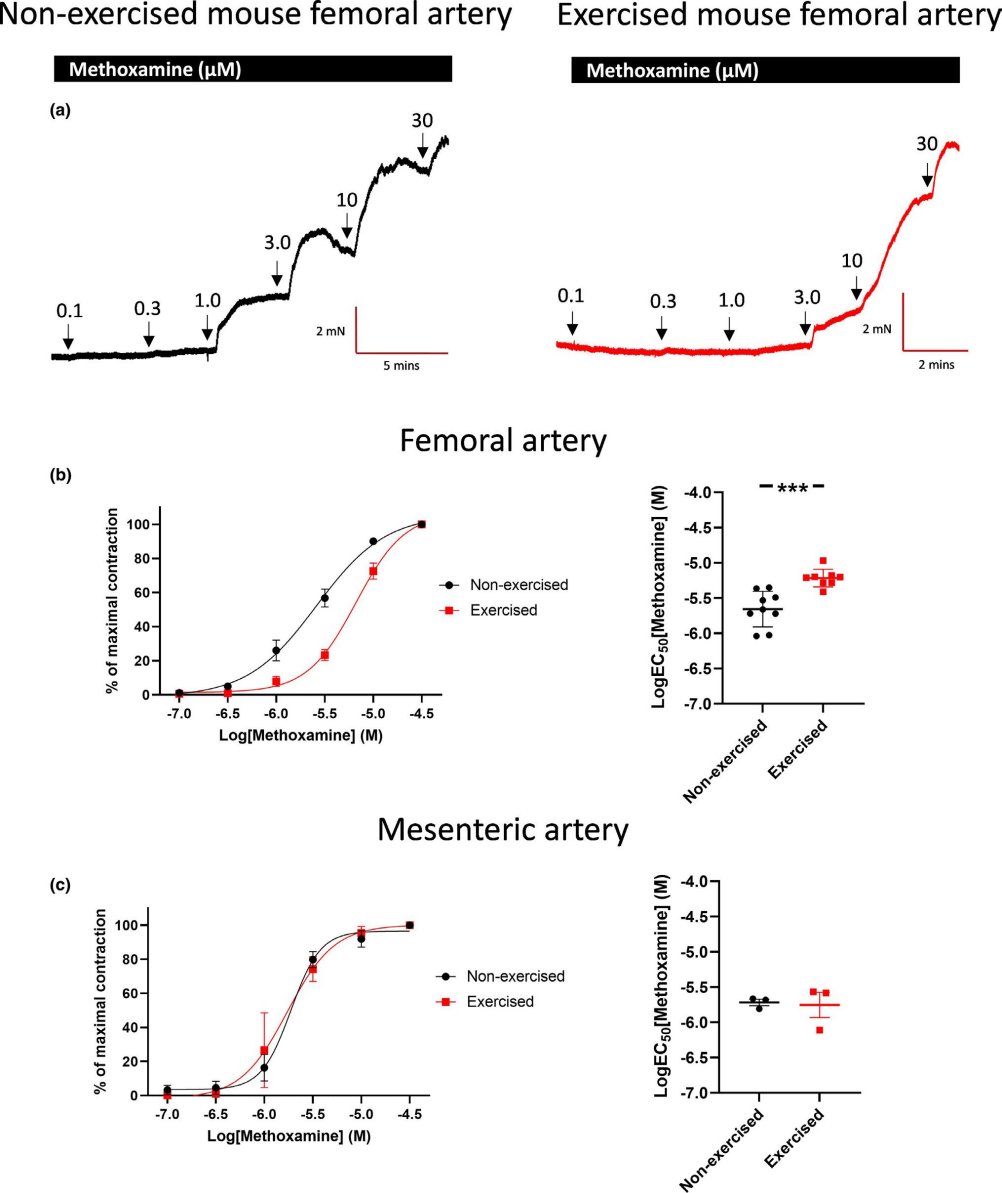
The vasoconstrictor response to methoxamine is blunted in ex vivo femoral arteries of exercised mice, but not affected by exercise in mesenteric arteries. (a) Representative isometric tension recordings of femoral artery segments from non‐exercised (left) and exercised (right) mice with increasing concentrations of α‐adrenergic receptor agonist methoxamine. (b) Mean concentration–effect curves and logEC_50_ values to methoxamine, showing reduced α‐adrenergic responsiveness in femoral arteries from exercised (*n* = 8) compared to non‐exercised (*n* = 9) mice. (c) Mean concentration–effect curves and logEC_50_ values to methoxamine, showing equal α‐adrenergic responsiveness in mesenteric arteries from non‐exercised (*n* = 3) and exercised (*n* = 3) mice. Statistical comparisons on the logEC_50_ values are performed with an unpaired *t* test. ***p < 0.0001. Data are presented as means ± SEM

Constriction of femoral artery segments to the α_1_‐adrenoceptor agonist, methoxamine, was attenuated in exercised mice (*n* = 8) compared to non‐exercised mice (*n* = 9), with mean logEC_50_ values of −5.2 ± 0.04 M and −5.7 ± 0.08 M (*p* < 0.001), respectively (Figure 1a,b). In contrast, the effect of methoxamine in mesenteric artery segments from exercised mice was not different from that of non‐exercised mice (*n* = 3; logEC_50_: −5.8 ± 0.18 and −5.7 ± 0.05 (*p* = 0.86), respectively; Figure 1c).

**FIGURE 2:**
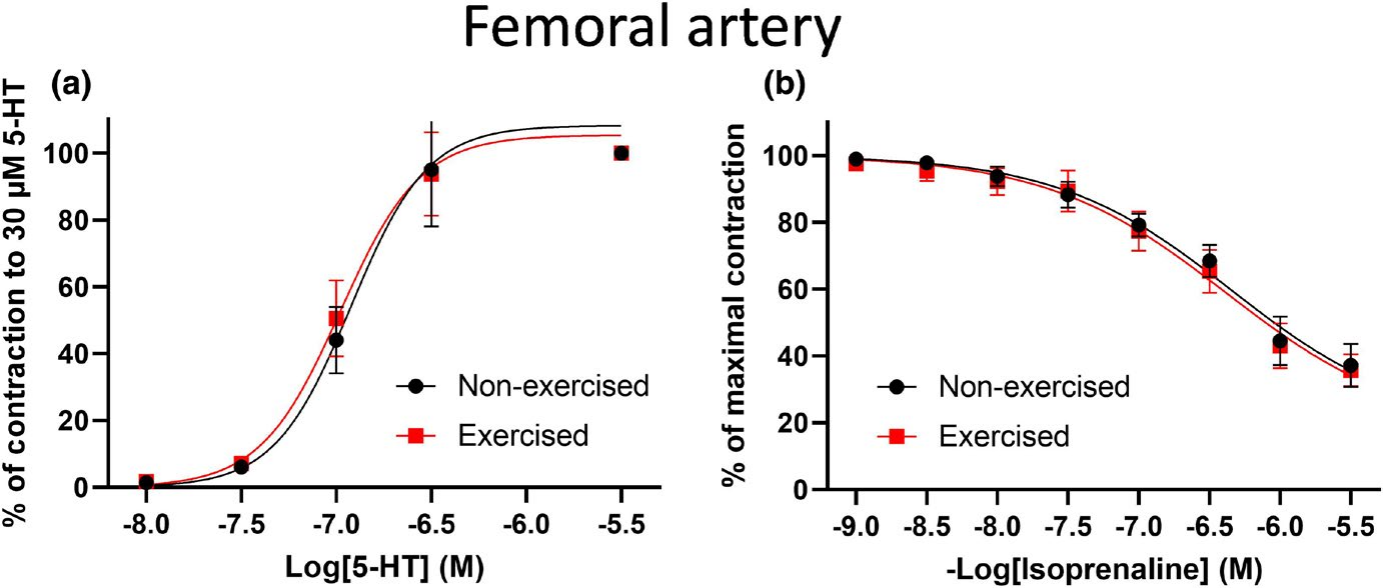
The response to 5‐HT and isoprenaline of ex vivo femoral artery segments is not affected by exercise. (a) Mean concentration–effect curves for 5‐HT in isometric tension recordings performed on femoral artery segments from non‐exercised (*n* = 3) and exercised (*n* = 4) mice. (b) Mean concentration–effect curves for the β‐adrenergic receptor activator, isoprenaline, after preconstriction of femoral artery segments from non‐exercised (*n* = 5) and exercised (*n* = 5) mice with methoxamine. Data are presented as means ± SEM

We next investigated the vasoconstrictor effects of 5‐HT in non‐exercised and exercise‐trained femoral artery segments. Non‐exercised and exercised femoral artery segments showed equal sensitivity to 5‐HT (*n* = 3–4; logEC_50_: −6.9 ± 0.11 and −6.9 ± 0.12; Figure 2a). Thus, exercise reduced the sensitivity to methoxamine, but not 5‐HT, in mouse femoral artery segments.

To test whether the sympatholytic effect of exercise was due to a shift toward more activation of β‐adrenergic receptors, and thus an enhanced vasodilation, we applied a β‐adrenergic receptor agonist, isoprenaline, to non‐exercised and exercised femoral artery segments. There was no difference in the isoprenaline‐mediated relaxations in femoral artery segments of non‐exercised and exercised mice (*n* = 5; *p* = 0.51; Figure 2b).

**FIGURE 3:**
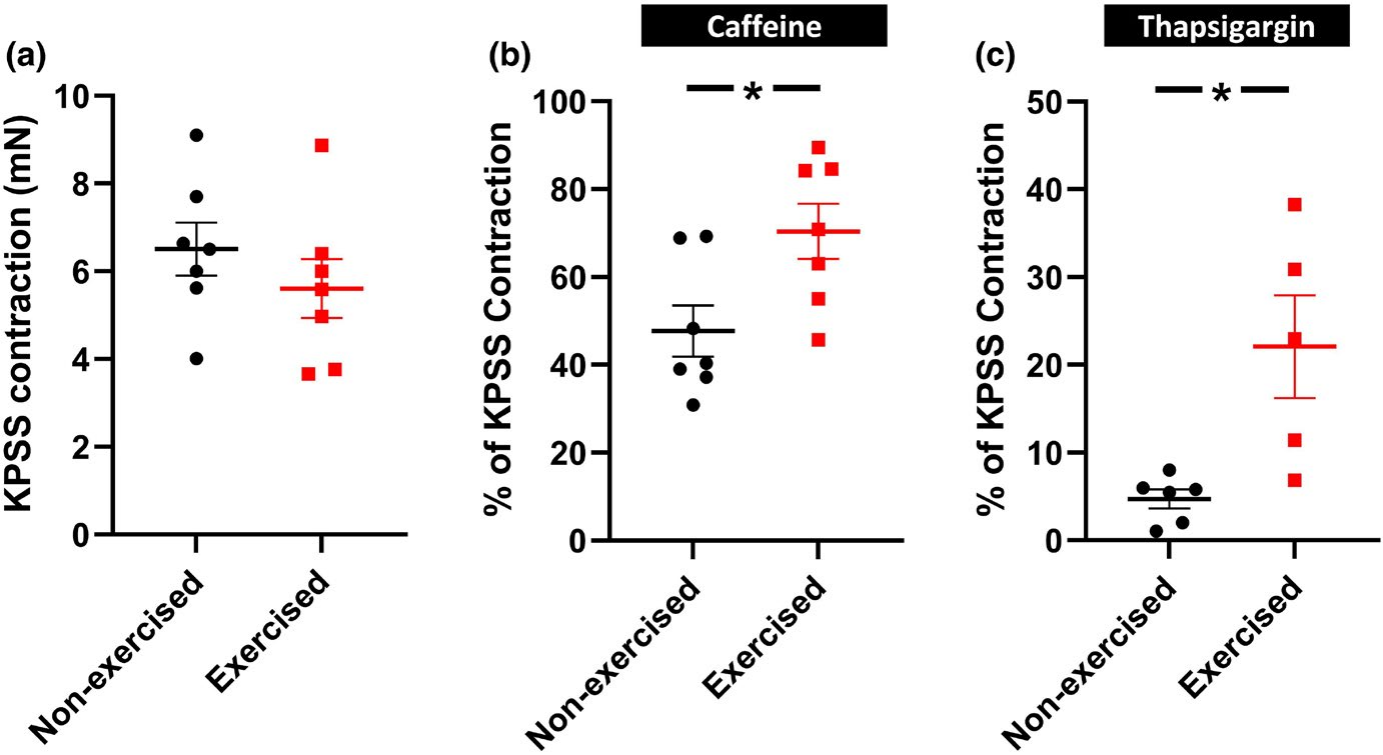
Femoral artery contractions induced by caffeine and thapsigargin are enhanced in ex vivo femoral artery segments from exercised mice. (a) Contractions to KPSS are equal in exercised and non‐exercised (control) mice (*p* = 0.34). (b) Mean contraction to 10 mM caffeine (ryanodine receptor agonist, *n* = 7) and (c) 1 μM thapsigargin (SERCA inhibitor, *n* = 5–6) in femoral artery segments from control and exercised mice (relative to 125 mM KCl response). Statistical comparisons on the contraction values are performed with an unpaired *t* test. * *p* < 0.05. Data are presented as means ± SEM

We next investigated potential differences in Ca^2+^ influx, and SR Ca^2+^ release and uptake properties in the femoral artery segments of the exercised and non‐exercised mice. Both exercised and non‐exercised femoral artery segments responded equally to contractions elicited by KPSS (*n* = 7; *p* = 0.34; Figure 3a). Contractions to caffeine (10 mM), which depletes SR Ca^2+^, were greater in the femoral arteries of exercised mice compared to the non‐exercised mice (*n* = 7; *p* = 0.02; Figure 3a), suggesting increased SR Ca^2+^ content. Furthermore, thapsigargin (1 μM), a selective inhibitor of the SR Ca^2+^‐ATPase (SERCA), induced larger constrictions in the femoral arteries of the exercised mice compared to the non‐exercised mice (*n* = 5–6; *p* = 0.011; Figure 3b). These results suggest increased SR Ca^2+^ content in the femoral artery of exercised mice.

**FIGURE 4:**
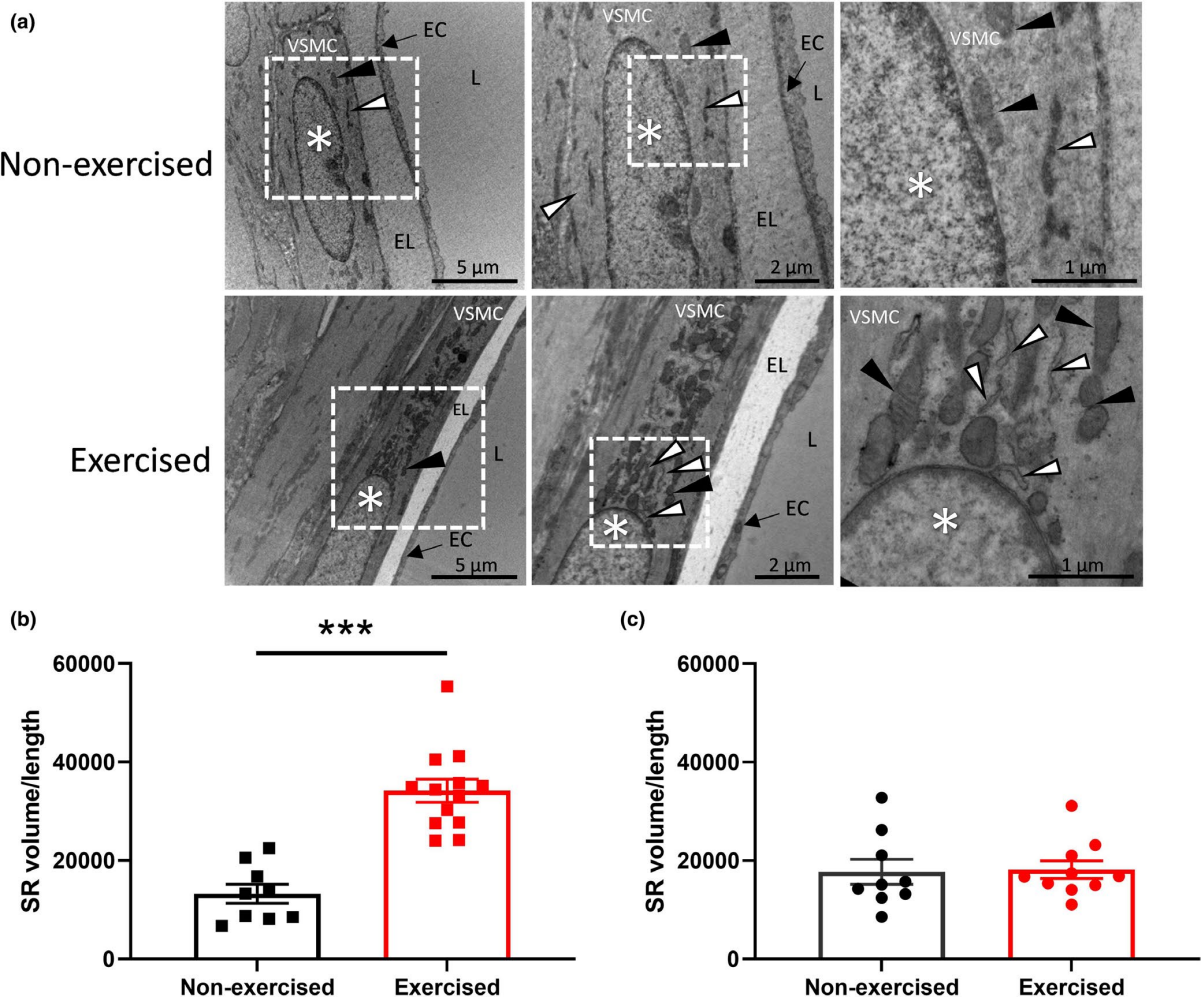
The volume of the sarcoplasmic reticulum is increased in arterial smooth muscle cells of the femoral artery from exercised mice compared to non‐exercised mice. (a) Transmission electron microscopy of a femoral artery segment from non‐exercised and exercised mice at increasing magnification (1–3), showing characteristic substructure of vascular smooth muscle cell particles. White arrows point to sarcoplasmic reticulum, black arrows point to mitochondria, and nuclei are indicated by the * symbol. L, vascular lumen; EC, endothelial cell lining, and EL, internal elastic lamina. Mean sarcoplasmic volume/length ratios in arterial smooth muscle cells from (b) femoral artery segments and (c) mesenteric artery segment of non‐exercised and exercised mice. SR is analyzed from femoral and mesenteric arteries taken from three animals, with 2–4 SR quantified per arterial section from each animal. Statistical comparisons on the SR volume/length values are performed with an unpaired *t* test. *p < 0.05. Data are presented as means ± SEM

To investigate changes in SR Ca^2+^ content, we performed 3D visualization of exercised and non‐exercised mouse femoral arteries with electron microscopy (FEI Teneo Volume Scope). Figure 4a shows representative electron microscopy images of a femoral artery segment from exercised and non‐exercised mice, showing characteristic substructure of vascular smooth muscle cells, including the SR. Quantification of the SR showed a 157% higher SR volume/length ratio in femoral arteries of exercised compared to non‐exercised mice (*p* < 0.0001; Figure 4b). In contrast, there was no significant difference in the SR volume/length ratio in mesenteric arteries of exercised versus non‐exercised mice (*p* = 0.88; Figure 4c). These results show that exercise results in swollen SR in the smooth muscle cells of femoral arteries, which is potentially due to increased SR Ca^2+^ content.

## 4 DISCUSSION

During exercise SNA increases, yet SNA‐mediated vasoconstriction is diminished in the arteries of working skeletal muscle. This was first described by Rein, 1930 and confirmed by Remensnyder et al., 1962, who termed the phenomenon “functional sympatholysis.” Although it is over 90 years since it was first described, to the best of our knowledge, this is the first study investigating the underlying mechanisms of functional sympatholysis in *isolated* arteries from exercised mice. Using this model, we observed the following: (1) the α‐adrenergic responsiveness is reduced in femoral artery segments of exercised mice after isolation from the working muscle; (2) caffeine‐induced contraction of femoral artery segments is increased in exercised mice; (3) inhibition of SR Ca^2+^ uptake increases constriction in femoral artery segments of exercised mice; and (4) SR volume/length ratio is increased in femoral arteries of exercised mice. These observations suggest that the smooth muscle cells of the artery wall undergo adaptations to attenuate post‐junctional α‐adrenergic receptor responsiveness, which potentially involves increasing smooth muscle cell SR Ca^2+^ content in arteries of the working skeletal muscle, thereby decreasing intracellular Ca^2+^ levels available for smooth muscle contraction.

In this study, we show attenuation of α‐adrenergic‐mediated vasoconstriction is maintained in femoral artery segments isolated from exercised mice, meaning the arterial segments are no longer in the presence of the vasoactive metabolites or SNA. These results confirm that functional sympatholysis can be observed in our ex vivo model following a single bout of exercise. It is important to note that this ex vivo approach does not negate the roles of multiple vasoactive compounds in functional sympatholysis, which are likely to be drivers of the arterial adaptations described in this study. Functional sympatholysis has been described previously in conduit arteries, including the brachial artery, and increased blood flow has been reported in femoral arteries during exercise (Iepsen et al., 2018; Jendzjowsky & Delorey, 2013; Mortensen et al., 2012, 2014; Nyberg & Hellsten, 2016). Since blood flow is markedly increased in the femoral artery during exercise, it is possible that increased shear stress augments endothelial‐derived nitric oxide release, which can stimulate SERCA activity in vascular smooth muscle cells (Clifford & Hellsten, 2004; Cohen et al., 1999; Pohl et al., 1986). Although for methodological reasons, our data are derived from conduit arteries, it is likely that a similar mechanism is present also in the microcirculation where the vascular resistance and, thus, blood flow to the active muscle is determined. Future studies will investigate whether this novel mechanism is shared in the resistance arterioles of working skeletal muscle where nitric oxide is also released by the muscle itself. As a control, we used isolated mesenteric artery segments, which are not in proximity to exercising skeletal muscle. In these arteries, we found no difference in the response to methoxamine between the non‐exercised and exercised mice, suggesting that the change in the femoral arteries was due to functional sympatholysis from the exercising muscle. This is the first evidence to show that arteries undergo physiological adaptations allowing functional sympatholysis to persist, at least transiently, in arteries isolated from the working muscle.

Although NE released by the sympathetic nervous system primarily binds to α‐adrenoceptors, NE may also bind to β‐adrenergic receptors and cause vasodilation, which may blunt the SNA‐mediated vasoconstriction (Kneale et al., 2000). In the present study, isoprenaline produced similar vasodilator responses in femoral artery segments from non‐exercised and exercised mice. Thus, our data does not support an exercise‐induced increase in β‐adrenoreceptor sensitivity as a mechanism underlying functional sympatholysis in ex vivo femoral arteries. Although it does not rule out the possibility that exercise may increase the concentrations of ligands that enhance β‐adrenergic vasodilation in vivo, this finding agrees with observations in previous rodent and human studies, which found no difference in the effect of propranolol on blood flow through exercised and rested skeletal muscle (Cooper et al., 2019; Hartling et al., 1980).

We also tested another neurotransmitter, serotonin (5‐HT), which has potent vasoconstricting properties, predominantly through 5‐HT_2A_ receptors. In contrast to methoxamine, femoral arteries from both non‐exercised and exercised mice showed equal sensitivity to 5‐HT. This finding confirms that maximal contractility of the exercised mice arteries is not affected, but rather supports the idea that the decreased exercise‐induced sympathetic constriction is directly related to altered α‐adrenergic receptor response.

The SR plays a central role in appropriate regulation of Ca^2+^ signaling, and is capable of Ca^2+^ uptake, storage, and release. The SR Ca^2+^ ATPase (SERCA) is a pump that transports Ca^2+^ from the cytoplasm into the SR (Stammers et al., 2015). Thus, SERCA pump activity lowers the cytoplasmic Ca^2+^ concentration while the SR Ca^2+^ concentration increases. In human and rodent skeletal muscle fibers, SERCA expression and activity changes with exercise training, depending on the muscle type and training intensity, resulting in altered SR Ca^2+^ handling properties (Duhamel et al., 2007; Green et al., 2003; Kubo et al., 2003; Morissette et al., 2014; Stammers et al., 2015; Viru, 1994). However, to the best of our knowledge, no one has studied the effects of exercise on SR Ca^2+^ handling properties in vascular smooth muscle cells. In this study, we demonstrate that SR Ca^2+^ release, induced by the ryanodine receptor agonist, caffeine, produced stronger contractions in the femoral artery segments from exercised compared to non‐exercised mice. These results indicate that more Ca^2+^ can be released from the SR in exercised femoral artery segments, likely due to a greater SR Ca^2+^ content. Moreover, we blocked SR Ca^2+^ uptake with thapsigargin, a selective inhibitor of SERCA. Inhibiting SERCA increased constrictions in femoral artery segments from exercised compared to non‐exercised mice, suggesting that SERCA activity might be increased to maintain a lower intracellular calcium concentration. These results suggest that exercise can increase SERCA‐mediated SR Ca^2+^ uptake in vascular smooth muscle cells, similar to skeletal muscle fibers. Such a mechanism would increase SR Ca^2+^ content, and thereby lower the free cytoplasmic Ca^2+^ concentration, which could explain the decreased vasoconstriction observed in functional sympatholysis. To confirm this, we performed 3D electron microscopy imaging of the arterial wall, and observed SR volume/length ratio in femoral arteries from exercised and non‐exercised mice. This result has further strengthened our hypothesis that SR Ca^2+^ content is increased in the vasculature of the exercising muscle. Overall, this study shows the first potential evidence of altered SR Ca^2+^ handling in vascular smooth muscle cells in exercise and provides a potential mechanism contributing to functional sympatholysis.

A limitation of this study is that we have not investigated the exercise‐dependent mechanisms driving increased SERCA activity in vascular smooth muscle cells in the arteries of exercising muscle. SERCA activity is affected by several pathways, many of which are associated with exercise, including changes in Ca^2+^, pH, ATP, phospholamban, thyroid hormone, adenosine monophosphate‐activated protein kinase (AMPK), and adiponectin (Stammers et al., 2015). Future studies will investigate which of these pathways are driving the changes in vascular smooth muscle cell SERCA activity during exercise. Nitric oxide represents a particularly interesting metabolite that could underlie the observations in this study given its strong association with functional sympatholysis and its ability to increase SERCA activity (Cohen & Adachi, 2006; Cohen et al., 1999). Another limitation of our study is we have not determined for how long these vascular changes persist, post‐exercise. In this study, all myography experiments were performed within 2 h post‐exercise. A time‐dependent analysis of the functional changes is required in future studies to determine how long the arteries maintain these physiological adaptions, and determine if this is an acute, short‐term phenomenon or a long‐term alteration in the arteries. Finally, this study lacks intracellular calcium imaging to conclusively prove that changes in vascular smooth muscle cell intracellular calcium levels underlie the attenuated methoxamine responses and that more calcium is stored in the SR and released upon caffeine treatment. Thus our interpretations of the current observations in this study must be reserved until we can perform these experiments.

In summary, our experimental approach provides a novel method for investigating functional sympatholysis, avoiding the continuous complex interplay between sympathetic vasoconstrictor outflow and vasoactive signals released locally from the muscle. We have demonstrated that α‐adrenergic responsiveness is attenuated in isolated femoral artery segments of exercised compared to non‐exercised mice, which is potentially due to increased Ca^2+^ uptake by the SR in vascular smooth muscle cells, although further experiments are required to fully define this mechanism. Based on the observations in this study, we suggest that functional sympatholysis involves fundamental changes in arterial smooth muscle cells, which contribute to the increase in blood flow to the working skeletal muscle.

## CONFLICT OF INTEREST

No conflict of interest, financial or otherwise, are declared by the authors.

## AUTHOR CONTRIBUTIONS

All authors were responsible for the experimental design and contributed to performing the experiments; T.A.J. and Y.H. conceived and designed the research; J.vdH., S.M., S.A.S.K., and T.A.J. performed the experiments; J.vdH. and T.A.J. analyzed the data; J.vdH., T.A.J., and Y.H. interpreted the results of experiments. J.vdH., S.M., T.A.J., and Y.H. drafted the manuscript. All authors contributed to the revision of the manuscript and have approved the final version prior to submission.
